# Efficacy of a topical formulation combining fipronil, moxidectin, and praziquantel (Banni3) in controlling flea infestation in cats

**DOI:** 10.3389/fvets.2025.1551961

**Published:** 2025-02-13

**Authors:** Diefrey Ribeiro Campos, Ester Oliozi Marré, Breno Cayeiro Cruz, Gessica Ariane de Melo Cruz, Milenni Garcia Michels, Igor Renan Honorato Gatto, Ferdinando Nielsen de Almeida, Dandara Quelho Rosa, Thais Ribeiro Correia, Fabio Barbour Scott

**Affiliations:** ^1^Laboratory of Experimental Chemotherapy in Veterinary Parasitology (LQEPV), Department of Animal Parasitology, Institute of Veterinary Medicine, Federal Rural University of Rio de Janeiro, Seropédica, Brazil; ^2^Ourofino Saúde Animal Ltda, São Paulo, Brazil

**Keywords:** *Ctenocephalides felis felis*, flea control, cat flea, feline, treatment

## Abstract

*Ctenocephalides felis felis* is the most common parasitic insect worldwide, responsible for transmitting pathogenic agents to cats. The aim of this study was to evaluate the efficacy of a novel formulation containing fipronil 12.5%, moxidectin 0.83%, and praziquantel 8.3%, in the treatment of cats, both artificially as well as naturally infested with *C. felis felis*. For the controlled study, 14 cats were divided into a control group that received no treatment and a treated group that received the investigational product (Banni3 – 12.5% fipronil + 0.83% moxidectin + 8.3% praziquantel – Ourofino Saúde Animal Ltda.). Each animal was artificially infested 48 h before each evaluation with 100 fleas. Evaluations to determine efficacy were based on the average count of live fleas found in the control and treated groups and were carried out nine times after treatment for 56 days. For the field study, 110 indoor cats, naturally infested with *C. felis felis*, were included. These animals were divided into the positive control group, which received a topical formulation of 10% fipronil, and the treated group which received the investigational product. The efficacy evaluations were based on the average number of live fleas found pre-treatment and post-treatment and were carried out on days four times after treatment 30 days. For artificially infested cats, there was a statistically significant difference (*P* < 0.05) between the mean flea counts of the negative control and treated groups, and the efficacy was 100% from the second day up to 14 days after treatment, and then 99.3%, 97.8%, 96.0%, 92.0% and 92.5%, respectively, 21, 30, 35, 42 and 49 days after treatment. For cats that had natural infestations, the effectiveness was 98.6%, 99.7%, 98.7% and 97.3% on days 7, 14, 21 and 30 after treatment, respectively. There was no statistically significant difference (*P* > 0.05) when comparing flea counts between the treated group and positive control, but there were statistically significant differences (*P* < 0.05) in the pre- and post-treatment evaluations. A topical treatment with Banni3, administered as a single dose, is proven safe and effective for controlling *C. felis felis* in artificially and naturally infested cats.

## 1 Introduction

The subspecies *Ctenocephalides felis felis* (Siphonaptera, Pulicidae) has a cosmopolitan distribution, and its preferred hosts are cats and domestic dogs (1). Due to the hematophagous habit of adults, their presence in the host is associated with blood loss, allergic dermatitis, intense itching, as well as the transmission of pathogenic agents, such as *Bartonella* and *Rickettsia* (2–4). Furthermore, *C. felis felis* can act as an intermediate host of *Dipylidium caninum*, a cestode that parasitizes the intestinal tract of domestic animals as well as humans (5).

Flea control in companion animals is currently carried out essentially using ectoparasiticidal drugs. There are currently several classes of these products on the world market that have proven activity against *C. felis felis*. Fipronil, a phenylpyrazole, has been used since the 90s and, despite reports of resistant *C. felis felis* populations in some locations around the world, is still one of the most effective compounds in controlling this ectoparasite (6, 7).

Multiparasitism is frequently reported in cats, and the association of ectoparasites (fleas or mites) with gastrointestinal helminths is common for these animals (8, 9). In this context, when thinking about the treatment of cats, the preference of owners for topically administered formulations, as they reduce animal handling, and also for products that contain combinations of antiparasitics, that increase the spectrum of action and reduce the number of treatments, is notable, as these products facilitate the management of these animals, also reducing stress and risk of physical harm to cats and owners (10). For this reason, the aim of this work was to demonstrate the effectiveness of a formulation containing fipronil (12.5%) + moxidectin (0.83%) + praziquantel (8.3%) (Banni3 – Ourofino Saúde Animal Ltda.) in controlling fleas in artificially and naturally infested cats.

## 2 Materials and methods

The two studies followed a controlled, randomized, and investigator-blinded protocol, being conducted in the Rio de Janeiro state, Southeast region of Brazil. Each included both a treated group (being administered Banni3 – 12.5% fipronil + 0.83% moxidectin + 8.3% praziquantel – Ourofino Saúde Animal Ltda.) and control groups (either a negative or a positive control). The studies took place either under field conditions or at the Laboratório de Quimioterapia Experimental em Parasitologia de Veterinária (LQEPV), from the Veterinary Institute of Universidade Federal Rural do Rio de Janeiro. The guidelines followed for determining the effectiveness of the tested formulation (Banni3 – Ourofino Saúde Animal Ltda.) on the controlled and field studies were those recommended by the World Association for the Advancement of Veterinary Parasitology (WAAVP) (11). All cats in this study were managed in accordance with the guidelines set forth by ISFM/AAFP (12).

### 2.1 Controlled test

#### 2.1.1 Animals

Sixteen healthy mixed breed cats participated in the study, being 10 females and six males, aged between 26 and 82 months. The selected animals were kept in individual cages throughout the study period. The use of animals was approved by the Ethics Committee on the Use of Animals of the Veterinary Institute of the Federal Rural University of Rio de Janeiro, with protocol number 3123210121.

#### 2.1.2 Flea origin and infestations

In this study, fleas of the subspecies *C. felis felis* were used, kept on cats in a laboratory colony,. For artificial infestations, cats were infested with 100 unfed, 14-day-old adult fleas (± 50 males and 50 females) in the dorsal region, being restrained for 1 min after infestation to prevent flea ingestion. Infestations occurred on days-7, for randomization, on day-2, pre-treatment and on days 5, 12, 19, 28, 33, 40, 47, and 54, post-treatment.

#### 2.1.3 Randomization

After preliminary flea counting carried out 5 days before treatment, the cats were divided, respecting the proportion of males and females, into two experimental groups: control and treated.

#### 2.1.4 Treatment

On the day of treatment (day 0), each animal from the treated group received the investigational product (Banni3 – 12.5% fipronil + 0.83% moxidectin + 8.3% praziquantel – Ourofino Saúde Animal Ltda.) topically, on the dorsal area, according to the weight and indicated dose (0.3 mL for animals up to 2.5 kg; 0.9 mL for animals from 2.6 to 7.5 kg). Cats in the control group did not receive any type of treatment.

#### 2.1.5 Parasites counts and efficacy determination

Flea recovery and counting were performed using the comb test, in which each cat was combed along its entire body length for at least 5 min, and/or until the fleas were completely removed. Any flea that showed any type of movement was considered alive. The procedure always occurred 48 hours after infestation, the first happening on day-5, for randomization of the animals, then weekly on days 7, 14, 21, 30, 35, 42, 49 post-treatment, with the last happening on day 56, for completion of the study.

The calculation of efficacy was based on the following formula: $Efficacy\left(\%\right)=\left(\frac{Mc-Mt}{Mc}\right)x\;100.$ In this formula, M_C_ and M_T_ are, respectively, the arithmetic means (M) of the live flea counts in the control (_C_) and treated (_T_) groups. Statistical analyses were performed using the Bioestat 5.3 program. The Shapiro Wilk test confirmed a normal distribution (*P* > 0.05) for the study sample group, based on the individual flea counts of each animal. Using the independent sample *t*-test, the flea counts of the treated and control groups were compared and summarized as arithmetic means and ranges, used to calculate efficacy. The significance level considered in all tests was 95% (*P* ≤ 0.05).

### 2.2 Field test

#### 2.2.1 Animals

For this study, 110 cats were selected, without distinction of sex or age, all naturally infested with at least 10 live fleas of the *C. felis felis* subspecies. All cats originated from owners located in the city of Rio de Janeiro, state of Rio de Janeiro, Southeast region of Brazil. To include the animal in the study, the owner signed the Informed Consent Form, authorizing the cat's participation throughout the test period. The use of the animals was approved by the Animal Use Ethics Committee of the Veterinary Institute of the Federal Rural University of Rio de Janeiro, under protocol number 6013141220.

#### 2.2.2 Randomization

The cats were distributed into the positive control group or the treated group, one by one, based on the order of inclusion of their residences in the study. If there was more than one cat in the residence, everyone was included in the same group (all cats from the same household were treated with the same product).

#### 2.2.3 Treatment

The treatment process for the treated group was conducted in accordance with the methodology described in the controlled test. Animals in the positive control group received a commercial topical formulation, containing 10% fipronil, in a single administration spot-on (0.5 mL per cat, as indicated by the manufacturer).

#### 2.2.4 Parasite counts and efficacy determination

After inclusion of the animal in the study and its respective treatment, new flea counts, with removal of parasites, occurred on days 7, 14, 21, and 30 after treatment. The animal combing procedure followed the same protocol described for the controlled test.

To determine efficacy, the following formula, indicated by Benchaoui et al. (13), was used: $Efficacy\;\left(\%\right)=\left[\frac{\left[MAT\left(D-7e\;D-2\right)-MDT\right]}{MAT\left(D-7\;e\;D-2\right)}\right]\times 100$. In this formula, MAT (D-7 and D-2) is the arithmetic mean of the number of live fleas pre-treatment on days-7 and−2, and MDT represents the arithmetic mean of the number of live fleas post-treatment for each evaluation. Statistical analysis was performed by Wilcoxon test to compare the average number of live fleas before and after treatment. The Maan-Whitney test was used to compare the average number of live fleas between the two groups. The significance level considered in all tests was 95% (*P* ≤ 0.05). Statistical analyses were performed using the free statistical computational program Bioestat 5.3.

## 3 Results

### 3.1 Controlled test

All cats that participated in this study were mixed breed and shorthair cats, with five females and two males in each group. The mean age and weight of the animals was 58.4 months and 3.6 kg for the treated group and 62.3 months and 3.7 kg for the control group (Table 1), with no statistically significant difference for these parameters between the groups. Furthermore, no adverse events were observed with the cats during the development of this study.

**Table 1:** General data on the cats that participated in the controlled study evaluating the efficacy of the investigational product (Banni3 – 12.5% fipronil + 0.83% moxidectin + 8.3% praziquantel – Ourofino Saúde Animal Ltda.) against *Ctenocephalides felis felis* in artificially infested cats.

**Group**	**Number of cats**	**Breed**	**Coat**	**Gender**		**Age (months)**	**Weight (Kg)**
Negative control	7	Mixed Breed	Short fur	F (*n* = 5) M (*n* = 2)	Mean (sd)	58.4 (24.6)	3.6 (0.7)
					Median	56	3.5
					Min–Max	27–82	2.7–4.7
Treated	7	Mixed breed	Short fur	F (*n* = 5) M (*n* = 2)	Mean (sd)	62.3 (24.9)	3.7 (0.7)
					Median	70	3.7
					Min–Max	26–82	2.5–4.5
					*P-value*	0,8480	0,9240

F, female; M, male; Min, minimum; Max, maximum; sd, standard derivation.

Regarding the counts of live fleas before treatment, it was observed that the average counts were 40.7 and 41.1 for the control and treated groups, respectively, with no statistical difference between them (*P* = 0.8555). From the second day after treatment, up to the 14th day post-administration (Banni3 – fipronil 12.5% + moxidectin 0.83% + praziquantel 8.3% – Ourofino Saúde Animal Ltda.), no live fleas were found on the animals in the treated group, demonstrating 100% efficacy of the combination during this period. In the assessments conducted 21, 30, 35, 42, and 49 days after treatment, efficacies of 99.3%, 97.8%, 96.0%, 92.0%, and 92.5%, respectively, were observed. A statistically significant difference was observed in all evaluations after treatment (*P* = 0.0017), when comparing both groups. The summarized data regarding live flea counts and the formulation's efficacy can be seen in Table 2.

**Table 2:** Mean counts of *Ctenocephalides felis felis* between control and treated groups, efficacy indexes, and bilateral *P* values on different evaluation days (pre-treatment and after treatment) in artificially infested cats taking part on a controlled study evaluating the efficacy of the investigational product (Banni3 – 12.5% fipronil + 0.83% moxidectin + 8.3% praziquantel – Ourofino Saúde Animal Ltda.).

**Group**		**Experimental days**									
		**-5**	+**2**	+**7**	+**14**	+**21**	+**30**	+**35**	+**42**	+**49**	+**56**
Negative control	Mean (sd)	40.7 (8.2)	39.4 (10)	39.7 (11)	38.4 (13.1)	42.7 (21.5)	45.1 (12.4)	43.3 (12.4)	37.6 (11.4)	51.7 (13.8)	37.7 (12.8)
	Median	42	42	38	42	36	49	43	33	53	37
	Min–Max	28–49	28–54	28–62	18–57	22–74	30–63	30−63	24–56	34–71	21–59
Treated	Mean (sd)	41.4 (6)	0	0	0	0.3 (0.5)	1.0 (1.2)	1.7 (1.8)	3.0 (2.0)	3.9 (2.3)	10.0 (4.2)
	Median	40	0	0	0	0	1	1	3	4	11
	Min–Max	36–52	0	0	0	0–1	0–3	0–5	0–6	0–7	4–15
	Efficacy (%)	- - -	100	100	100	99.3	97.8	96.0	92.0	92.5	73.5
	*P-value*	0.8555	0.0017	0.0017	0.0017	0.0017	0.0017	0.0017	0.0017	0.0017	0.0010

Negative control group = cats without treatment; Treated group = cats that have received topical administration of the investigational product (Banni3 – 12.5% fipronil + 0.83% moxidectin + 8.3% praziquantel – Ourofino Saúde Animal Ltda.); sd, standard derivation; min, minimum; max, maximum; P value comparing tick counts among groups.

### 3.2 Field test

A total of 110 cats were included in the field study, of which 48 were treated with the positive control, and 62 were treated with the investigational product (Banni3 – 12.5% fipronil + 0.83% moxidectin + 8.3% praziquantel – Ourofino Saúde Animal Ltda.). The summarized data on the animals that participated in the study are shown in Table 3.

**Table 3:** General data on the cats that participated in the field study evaluating the efficacy of the investigational product (Banni3 – 12.5% fipronil + 0.83% moxidectin + 8.3% praziquantel – Ourofino Saúde Animal Ltda.).

**Group**	**Number of cats**	**Breed**	**Coat**	**Gender**		**Age (months)**	**Weight (Kg)**
Positive Control	48	MB (*n* = 45) Siamese (*n*= 3)	Short fur (*n* = 31) Middle fur (*n* = 11) Long fur (*n* = 6)	F (*n* = 29) M (*n* = 29)	Mean (SD)	42.1 (25.1)	3.8 (0.9)
					Median	36	3.6
					Min–Max	8–108	2.1–5.5
Treated	62	MB (*n* = 61) Siamese (*n* =1)	Short fur (*n* = 46) Middle fur (*n* = 5) Long fur (*n* = 11)	F (*n* = 30) M (*n* = 32)	Mean (SD)	37.2 (28.5)	3.5 (0.8)
					Median	36	3.3
					Min–Max	5–96	2–6
					*P-value*	0.8545	0.9568

MB, mixed breed; F, female; M, male; Min, minimum; Max, maximum; sd, standard derivation.

The average counts of live fleas were 35 and 31 for experimental day-7, and 33.9 and 30.5 for experimental day-2. There was no statistical difference (*P* > 0.05) between the two experimental groups in the pre-treatment evaluations. For the positive control group, the efficacy indexes were 98.7%, 99.5%, 98.6%, and 96.6% for experimental days 7, 14, 21, and 30 post-treatments, respectively. Significant statistical differences (*P* < 0.0001) were observed when comparing the pre- and post-treatment averages in the positive control group. For the treated group, the efficacy indexes were 98.6%, 99.7%, 98.75, and 97.3% for experimental days 7, 14, 21, and 30 post-treatment, respectively, and significant statistical differences (*P* < 0.0001) were observed when comparing the pre- and post-treatment averages within the treated group. When comparing the average recovery of live fleas between the two experimental groups, no significant statistical differences were observed (*P* > 0.05). The summarized efficacy data from this study can be seen in Table 4.

**Table 4:** Mean counts of *Ctenocephalides felis felis* between control and treated groups, efficacy indexes, and bilateral *P* values on different evaluation days on pre-treatment and after treatment in naturally infested cats taking part on a controlled study evaluating the efficacy of the investigational product (Banni3 – 12.5% fipronil + 0.83% moxidectin + 8.3% praziquantel – Ourofino Saúde Animal Ltda.).

**Group**		**Experimental days**					
		**-7**	**-2**	**7**	**14**	**21**	**30**
Positive control	Mean (sd)	35 (16.5)	33.9 (15.9)	0.4 (1)	0.2 (0.5)	0.5 (1.1)	1.2 (2.1)
	Median	32.5	30.5	0.0	0.0	0.0	0.0
	Min–Max	12–79	10–78	0–5	0–2	0–7	0–10
	Efficacy	—	—	98.7	99.5	98.6	96.6
	*P-value* Intragroup	—	—	< 0.0001	< 0.0001	< 0.0001	< 0.0001
Treated	Mean (sd)	31 (15.8)	30.5 (15.5)	0.4 (1.2)	0.1 (0.4)	0.4 (1.4)	0.8 (2.3)
	Median	29	25.5	0	0	0	0
	Min–Max	10–74	12–88	0–6	0–3	0–7	0–15
	Efficacy (%)	—	—	98.6	99.7	98.7	97.3
	*P-value* Intragroup	—	—	< 0.0001	< 0.0001	< 0.0001	< 0.0001
	*P-value* Intergroup	0.6458	0.8765	0.8245	0.9941	0.5692	0.5154

Positive control = cats that have received a topical formulation containing fipronil 10%; Treated Group = cats that have received topical administration of the investigational product (Banni3 – 12.5% fipronil + 0.83% moxidectin + 8.3% praziquantel – Ourofino Saúde Animal Ltda.); min, minimum; max, maximum; sd, standard derivation; P value comparing fluea counts between the experimental groups (Positive Control Group vs. Treated Group) and among the groups.

## 4 Discussion

The investigational formulation (Banni3 - Ourofino Saúde Animal Ltda.), containing fipronil 12.5%, moxidectin 0.83%, and praziquantel 8.3% showed high efficacy in controlling *C. felis felis*, with maximum efficacy being registered as soon as 48 h after a single application. This is the first report of the combined use of these compounds for flea control. The preventive efficacy of fipronil was remarkable during the analyzed period, remaining above 96% up to 30 days after treatment in both tests. According to Carlotti and Jacobs (14), efficacy values must exceed 90% to ensure the parasiticidal activity of a compound. This index was reached up to 49 days post-treatment, on the trial involving artificially infested animals. Controlled studies with fleas are essential for evaluating the efficacy and persistence of antiparasitic products, as they allow for the control of environmental variables and precise analysis of treatment duration (15, 16). Furthermore, although this study did not evaluate the speed of flea elimination, Cruthers et al. (17) demonstrated that the *C. felis* flea can be 100% eliminated from previously infested dogs with the use of fipronil applied topically in the form of a spot-on, within 12–18 h after treatment, while according to Schenker et al. (18) after 8 h of topical application of fipronil in dogs, also previously infested, the average efficacy was 97.4% in controlling *C. felis*. For naturally infested dogs, Dryden et al. (19) reported that a single topical application of fipronil 10% w/v was 97% effective in reducing flea populations for 28 days, a value similar to that found in the present study, leading to a 96.5% reduction in the parasite load present in the animal.

The efficacy values in the field study were lower than those found in the controlled study and the formulation (Banni3 – Ourofino Saúde Animal Ltda. – fipronil 12.5% + moxidectin 0.83% + and praziquantel 8.3%) did not reach 100% efficacy in this trial. However, it is important to remember that field studies are conducted precisely to evaluate a new product/formulation in real (uncontrolled) conditions. In this way, abiotic factors such as luminosity and humidity can interfere with the product's efficacy. Additionally, intrinsic factors in cats, such as product removal during grooming or faster metabolism (which can vary among individuals), may also play a role. Furthermore, it is important to note that in these types of studies, environmental infestations tend to exist and can be intense, leading to a recurring challenge. Live fleas found on experimental days may result from new individuals that have just jumped onto the animals (20–22). However, it is important to emphasize that even though it did not achieve 100% efficacy, in all experimental days it was >97%.

The results obtained in these studies, with 49 days of sufficient efficacy indexes (100% efficacy starting on the second day after treatment; >99% efficacy up to day 21 [maximum efficacy up to day 14 on the controlled trial]; >95% efficacy up to day 35; >92% up to day 49), are similar to those found by Delcombel et al. (23), who achieved 93% efficacy after 4 weeks of using Frontline^®^ (fipronil) topically for the control of *C. felis felis* in a field study with dogs; by Champpell e*t al*. (21), who achieved 95.2% efficacy after 42 days of using fipronil in combination with dinotefuran topically in cats artificially infested with *C. felis felis*; and by Tancred e*t al*. (24), who obtained 89% efficacy at 28 days post-treatment in a controlled study using 10% fipronil topically for the control of *C. felis felis* in cats.

The insecticidal efficacy of this formulation against flea infestations in cats is due to the presence of fipronil, a phenylpyrazole widely used in the control of parasitic insects since the 1990s worldwide (25). The combination of moxidectin and praziquantel in the formulation is designed to increase the spectrum of action, allowing a single treatment to be effective against insects, mites, nematodes, and cestodes. This feature makes it appealing for cat owners who prefer to administer topical products instead of oral ones, as it is easy to apply and causes less stress for the animals, reducing the risk of physical harm to both the owner and the cat (26–28). Based on the information presented, the investigational formulation, Banni3 (Ourofino Saúde Animal Ltda.), containing fipronil 12.5% + moxidectin 0.83% + praziquantel 8.3%, is effective in controlling *C. felis felis*, with good efficacy guaranteed over a period of 30 days, providing protection for cats against the harmful effects of infestations and diseases transmitted by fleas while offering convenience for the owner in product application and frequency of use.

## 5 Conclusion

Based on the results obtained through the adopted experimental designs, it can be confirmed that the investigational product Banni3, composed of 12.5% fipronil + 0.83% moxidectin + 8.3% praziquantel, from the company Ourofino Saúde Animal Ltda., administered topically and in a single dose, exhibits, for 49 days after administration, over 90% efficacy against *C. felis felis* in artificially and naturally infested cats.

## Data Availability

The original contributions presented in the study are included in the article/supplementary material, further inquiries can be directed to the corresponding author.
